# Tungsten Doped TiO_2_ with Enhanced Photocatalytic and Optoelectrical Properties via Aerosol Assisted Chemical Vapor Deposition

**DOI:** 10.1038/srep10952

**Published:** 2015-06-04

**Authors:** Sanjayan Sathasivam, Davinder S. Bhachu, Yao Lu, Nicholas Chadwick, Shaeel A. Althabaiti, Abdulrahman O. Alyoubi, Sulaiman N. Basahel, Claire J. Carmalt, Ivan P. Parkin

**Affiliations:** 1 Materials Chemistry Centre, Department of Chemistry, University College London, 20 Gordon Street, London WC1H 0AJ, UK; 2 Bio Nano Consulting Ltd, The Gridiron Building, One St. Pancras Square, London N1C 4AG, UK; 3 Chemistry Department, King Abdulaziz University, Saudi Arabia; 4 Surface Chemistry and Catalytic Studies Group, King Abdulaziz University, Saudi Arabia

## Abstract

Tungsten doped titanium dioxide films with both transparent conducting oxide (TCO) and photocatalytic properties were produced *via* aerosol-assisted chemical vapor deposition of titanium ethoxide and dopant concentrations of tungsten ethoxide at 500 °C from a toluene solution. The films were anatase TiO_2,_ with good *n*-type electrical conductivities as determined *via* Hall effect measurements. The film doped with 2.25 at.% W showed the lowest resistivity at 0.034 Ω.cm and respectable charge carrier mobility (14.9 cm^3^/V.s) and concentration (×10^19^ cm^−3^). XPS indicated the presence of both W^6+^ and W^4+^ in the TiO_2_ matrix, with the substitutional doping of W^4+^ inducing an expansion of the anatase unit cell as determined by XRD. The films also showed good photocatalytic activity under UV-light illumination, with degradation of resazurin redox dye at a higher rate than with undoped TiO_2_.

Transparent conducting oxide (TCO) films have important applications in many opto-electronic devices such as solar cells and display technologies^1^,^2^,^3^,^4^. They combine electrical conductivity with optical transparency (>80% in the visible region)^1^,^5^. Traditional TCO materials are based around Sn doped In_2_O_3_ (ITO), however due to the high costs associated with indium, research has been focused on finding alternative materials with comparable conductivity and transparency to ITO^4^.

Titanium dioxide is a widely used intrinsically *n*-type semiconductor with primary applications in photocatalysis^6^,^7^. Recently, the use of TiO_2_ as a TCO material has gained widespread attention due to advantageous electronic properties and conductivity arising from *d* electrons unlike ITO where conductivity is due to *s* electrons^8^,^9^,^10^.

Furubayashi *et al.* has shown the possibility of achieving TiO_2_ thin films with respectable conductivities and transparencies using Nb and Ta as dopants using PLD^11^,^12^. Parkin *et al.* were able to produce Nb and Ta doped TiO_2_ films *via* CVD methods with sheet resistances of 6.5 Ω sq^−1^ and 14 Ω sq^−1^ respectively as well as resistivites of 1.1 × 10^−3^ Ω.cm and 2.7 × 10^−3^ Ω.cm^9^,^13^.

Using W as a dopant is another possibility as it enables the release of up to two electrons for every one dopant atom^10^,^14^. This is advantageous as it allows the use of low dopant levels that reduce defect concentrations and hence reduce scattering of charge carriers. W is an ideal dopant that is soluble in a TiO_2_ matrix because it commonly occurs in the 6+ oxidation state and in that state has a smaller ionic radius than Ti^4+^.^10^,^14^ Hence, there is potential to obtain better conductivity and transparency compared to Nb and Ta. Xu and Chen *et al.* were able to obtain resistivities in the order of 10^−2^ Ω.cm *via* magnetron co-sputtering^10^.

Furthermore, W doping of TiO_2_ has multiple advantages, as it is known to enhance the photocatalytic activity by reducing charge carrier recombination and by increasing light absorption by absorbing in the visible portion of the spectrum^15^.

In this paper we present the first synthesis of W doped TiO_2_ films *via* a specialized solution based chemical vapor technique (CVD) known as aerosol assisted (AA) CVD^9^,^16^,^17^,^18^. It is a simple, versatile and easily scalable technique that involves the transportation of the precursors into the deposition chamber in the form of aerosol droplets^19^. The films were deposited using a toluene solution of [Ti(OEt)_4_] and [W(OEt)_6_] on glass susbstrates at 500 °C and the films were transparent, photocatalyticically active, coloured and conducting - enabling four functional properties in the same film.

## Results and Discussion

Thin films of undoped and W doped TiO_2_ were deposited on glass substrates *via* AACVD at 500 °C using nitrogen as the carrier gas. An undoped TiO_2_ film was made from a toluene (25 mL) solution of [Ti(OEt)_4_], while for the W doped TiO_2_ films dopant concentrations (2–20 mol.% in solution) of [W(OEt)_6_] were used. This is to our knowledge the first example of W doped TiO_2_ being formed by AACVD. All films were well adhered to the substrate passing the Scotch^TM^ tape test. The undoped films were optically transparent and colourless where as the doped films were translucent blue, with the colour intensity increasing with increasing dopant concentration. The films were air stable, showing no change in colour or conductivity after 3 months.

**Figure 1:**
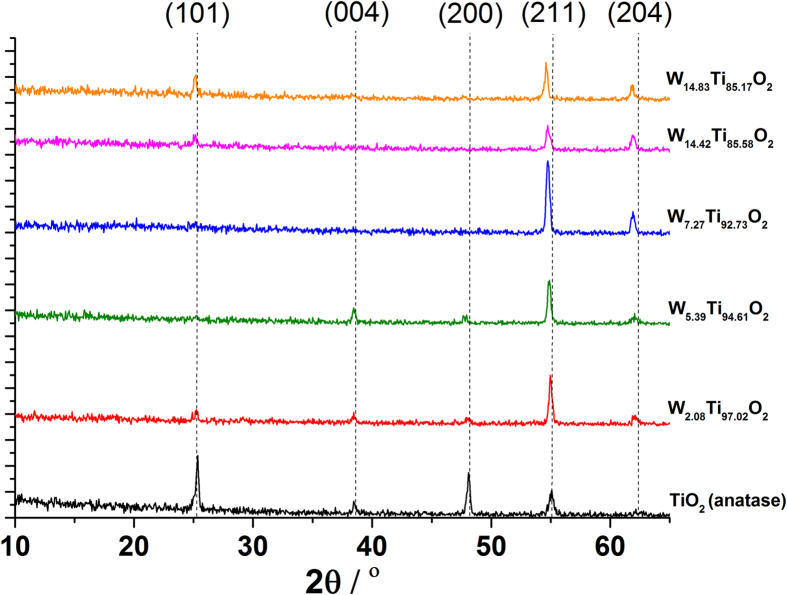
The XRD pattern for the undoped and W doped TiO_2_ films grown *via* AACVD from a toluene solution of Ti(OEt)_4_ and W(OEt)_6_ at 500 °C. The shift in the peaks to lower 2θ values upon doping with W is indicative of W^4+^ incorporation into the TiO_2_ host.

The XRD patterns for the undoped and W doped films show reflections matching the anatase phase of TiO_2_ (Fig. 1). The undoped film showed peaks corresponding to (101), (004), (200), (211) and (204) planes at 25.3°, 38.7°, 48.2°, 55.1° and 62.4° 2θ respectively. The doped samples showed a degree of preferred orientation in the (211) and (204) directions. This has been previously observed for metal (Nb and Ta) doped TiO_2_ films deposited using both AACVD and PVD^9^,^13^.

Due to the similarities in the ionic radii of W^6+^ (0.60 Å) and Ti^4+^ (0.605 Å), the substitutional doping of W^6+^ with Ti^4+^ should impart almost no change in the TiO_2_ unit cell. However, XPS results (see below) show that doped films contain W in both the 4+ and 6+ oxidation state. Therefore the observed linear shift of the XRD peaks to lower 2θ values, thus indicating an expansion in the TiO_2_ unit cell, was due to the partial replacement of Ti with W^4+^ that has an ionic radius of 0.66 Å. This follows closely with observations made by Kafizas and Parkin for W doped TiO_2_ system studied *via* combinatorial atmospheric pressure CVD where the majority of the W was in the 5+ oxidation state (0.62 Å)^15^. The lattice parameters for the undoped and doped films were calculated by fitting a La Bail model to the XRD data using GSAS and EXPGUI programs (Table 1).

**Figure 2:**
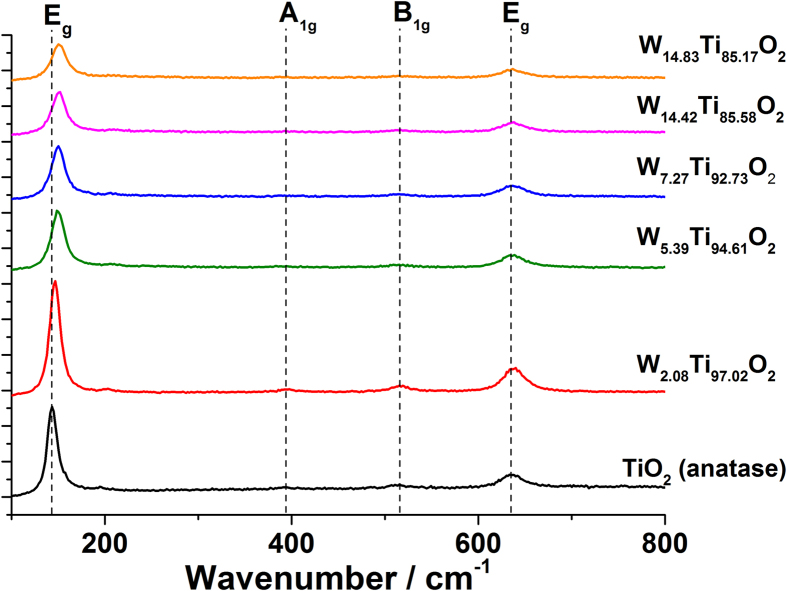
The Raman spectra for the undoped TiO_2_ and W doped TiO_2_ films grown *via* AACVD. A linear blue shift in the E_g_ peak (from 143–158 cm^−1^) that was observed is indicative of an expansion in the anatase unit cell.

Raman patterns taken for the undoped and W doped TiO_2_ films showed peaks expected for TiO_2_ anatase with tetragonal symmetry (Fig. 2)^20^. Upon doping of TiO_2_ the main peak (E_g_ vibrational mode) was shifted to higher wavenumbers indicating an expansion of the unit cell. The shift in the Eg peak increased linearly with increasing W concentration and is consistent with formation of a solid solution as observed by the XRD data.

Wavelength dispersive X-ray spectroscopy was used to determine the amount of W present in the films. The results show a linear increase in W content before possible saturation is reached at 15 mol.% of W in the AACVD precursor solution and 4.5 at.% in the films (Table 1).

**Figure 3:**
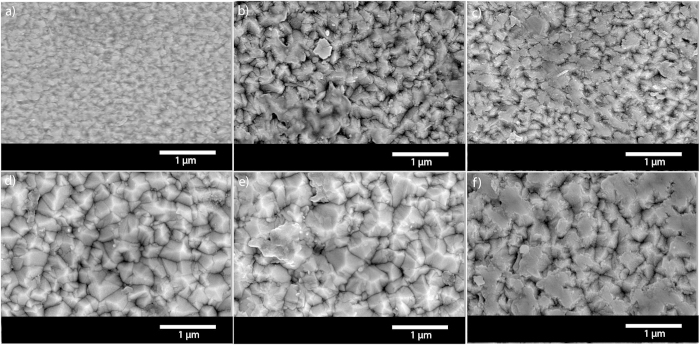
SEM micrographs of the AACVD grown W doped TiO_2_ films. The undoped film (**a**) shows typical morphology consisting of compact domes. Where as the doped films – 0.63 at.% (**b**), 1.65 at.% (**c**), 2.25 at.% (**d**), 4.47 at.% (**e**) and 4.65 at.% (**f**) W concentration - have a more structured morphology consisting of pyramidal features protruding perpendicular to the substrate.

Analysis of the film morphology using scanning electron microscopy (SEM) reveal that the surface of the AACVD grown films become more structured upon doping with W (Fig. 3). The morphology of the undoped TiO_2_ film consists of compact domes roughly 100 nm in width that transform to pyramidal features perpendicular to the substrate upon W incorporation. With increasing dopant concentration the features coalesce to form larger pyramids that are roughly 500 nm in width. Furthermore, the 4.47 W at.% and 4.65 W at.% films seem to show the features coalescing even further. This has been observed previously when other transition metals such as Nb and Ta have been substituted into a TiO_2_ matrix^9^,^13^. Film thickness, obtained *via* side-on SEM, show that in general there is an increase in the thickness with increasing amount of dopant. This coupled with the increase in film roughness contributes to a decrease in film transparency.

**Figure 4:**
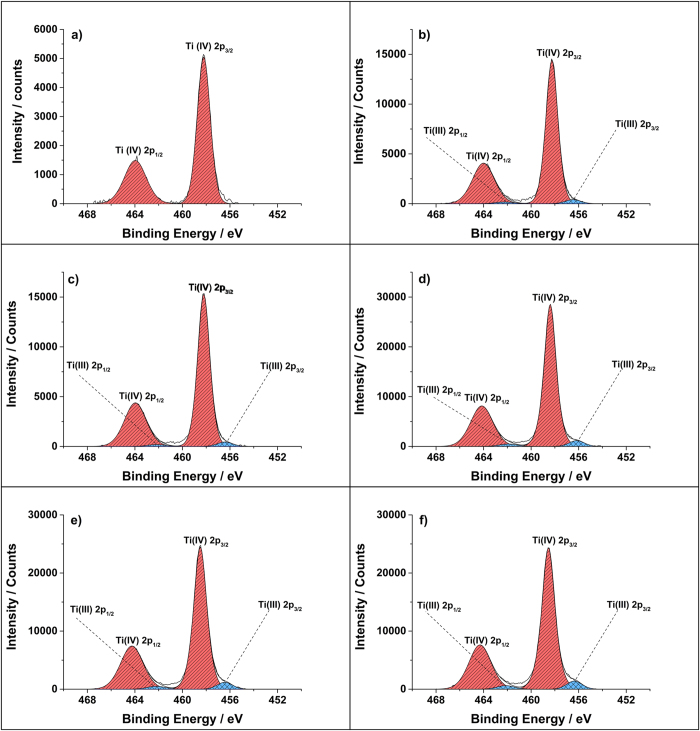
The XPS data for the undoped (**a**) and W doped TiO_2_ (**b**-**f**) films grown *via* AACVD at 500 °C. There is an increase in the Ti^3+^ peak intensity with increasing W concentration.

**Figure 5:**
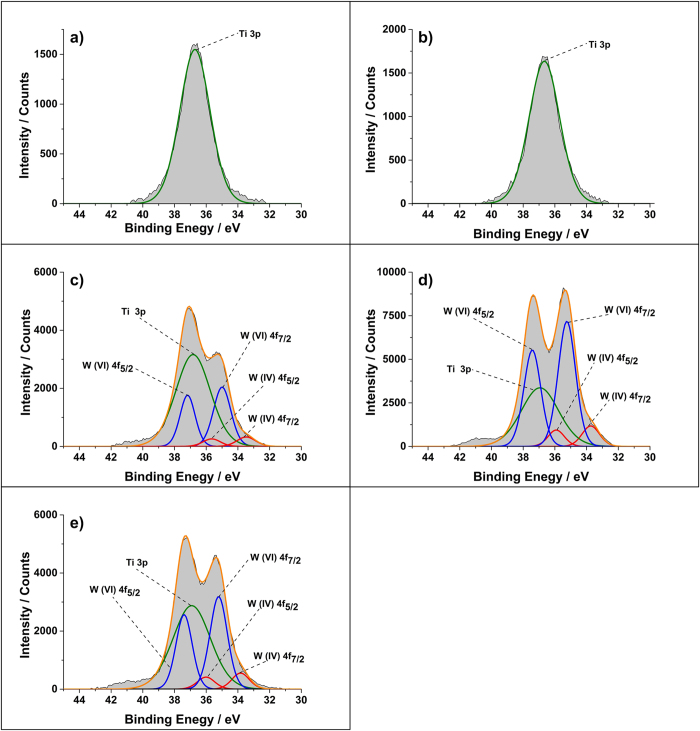
The Ti 2p and W 4f XPS peaks for the W doped TiO_2_ grown *via* AACVD. The intensity of the W 4f peak that coincides at the same region as Ti 2p increases with increasing dopant concentration. Both W 6+ and 4+ is observed.

X-ray photoelectron spectroscopy (XPS) was performed on the films to determine the oxidation state of the Ti and W species (Fig. 4 and Fig. 5). For the undoped film a 2p_3/2_ peak corresponding to Ti in the 4+ oxidation state was observed at 458.3 eV that matches to literature values^21^,^22^. The W doped TiO_2_ samples also showed this peak at 458.2 eV for Ti^4+^, but an additional 2p_3/2_ peak at 457.0 eV corresponding to Ti^3+^ was also observed^9^,^13^,^23^. The intensity of the Ti^3+^ peak increased with increasing W concentration. Thus indicating that the charge compensation by electrons for the substitutional doping of W 4+ and/or 6+ species for Ti^4+^ results in the reduction of the titanium to the 3+ oxidation state.

Peaks corresponding to W 4f overlap with Ti 3s peaks in XPS, therefore W was only detectable for the 2.25 at.% and above doped films (Fig. 5). In these films, 4f_7/2_ peaks corresponding to W^6+^ was seen at 35.2 eV as well as 4f_7/2_ peaks for W^4+^ at 33.8 eV^24^,^25^. There was consistently six times greater W^6+^ compared to W^4+^ in all the films. In a previous study where Kafizas and Parkin studied W doping of TiO_2_ *via* a combinatorial APCVD method, XPS studies showed the presence of mainly W^5+^ and some W^4+^, lacking the desired 6+ oxidation that enables enhanced electrical properties^15^.

The electrical properties of the doped films were determined though Hall effect measurements with all films displaying *n*-type conductivity (Table 2). The undoped sample was too resistive to measure on the Hall effect instrument but two-point probe measurements showed conductivity in the MΩ range. With a dopant concentration of 0.63 at.% and 1.65 at.% sheet resistance drops to 4.9 kΩ sq^−1^ and 3.4 kΩ respectively. For the 0.63 at.% doped sample the resistivity was 0.63 Ω.cm and the charge carrier concentration was to the order of 10^17^ cm^−3^ enabling a carrier mobility of 13 cm^2^/V.s. The resistivity (0.29 Ω.cm), charge carrier concentration (10^18^ cm^3^) and mobility (13 cm^2^/V.s) were also similar for the 1.65 at.% W doped film. A further increase in dopant concentration to 2.25 at.% resulted in the film with the best electrical properties for this study. There was a decrease in sheet resistance and resistivity to 212 Ω.sq^−1^ and 0.034 Ω.cm respectively. This is comparable to the results obtained by Xu and Jin *et al.* for W doped TiO_2_ films grown by rf sputtering. The carrier mobility, at 14.9 cm^2^/V.s, was slightly better than the 0.63 at.% and 1.65 at.% films while the carrier concentration was an order of magnitude higher. At 4.47 at.% doping of W in TiO_2,_ the sheet resistance was reduced to 78 Ω.sq^−1^ with a resistivity of 0.042 Ω.cm and an increased carrier concentration in the order of 10^20^ cm^−3^. However, the carrier mobility was diminished to 0.32 cm^2^/V.s (Fig. 3), this is likely to be due to the increased dopant amounts, specifically W in the 4+ oxidation, that behave as scattering sites for the charge carriers. In general, the lower than expected electrical performance of the W doped films synthesisied *via* AACVD is primarily due to the W^4+^ center reducing carrier mobility.

**Figure 6:**
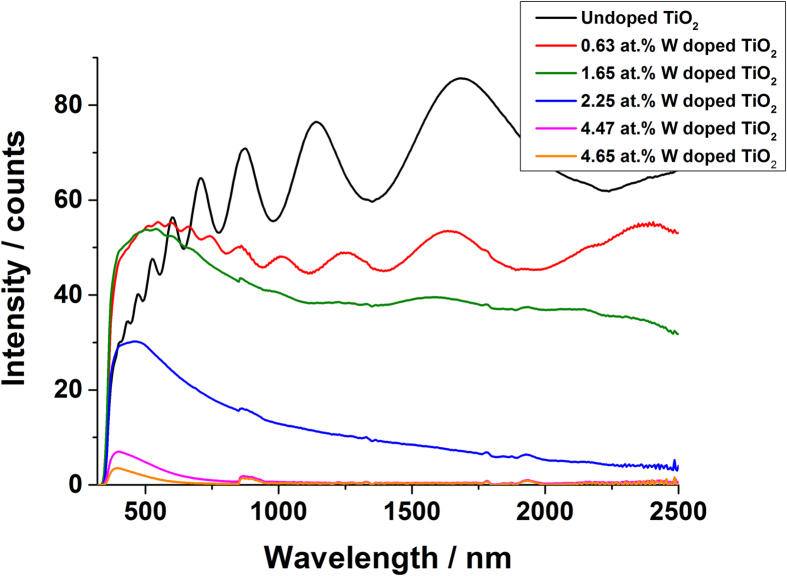
The UV-Vis transmission data for the W doped TiO_2_ films grown from the AACVD reaction of Ti(OEt)_4_ and W(OEt)_6_.

UV-Vis spectroscopy was used to show the optical transmission of the undoped and doped TiO_2_ films (Fig. 6). The undoped film was transparent in the visible region with 55% transmission, this was similar to the 0.63 at.% doped film that also showed transmission close to 55%. With increasing dopant concentration, the films become less transparent - transmission at a wavelength of 500 nm was roughly 50%, 30%, 7% and 3% for the 1.65 at.%, 2.25 at.%, 4.47 at.% and 4.67 at.%. The decrease in transmission has been observed previously for other transition metal doped TiO_2_ films and is attributed to the increase in absorption in the infrared region (see supporting information for reflectance and absorption curves)^9^,^13^. Furthermore, the transmission of the films decreases from 600 nm most likely due to Ti^3+^ states induced by the W doping that give rise to absorption from d-d transitions.

The UV light induced photocatalytic properties of the undoped and W doped TiO_2_ films were determined using resazurin dye based ‘intelligent ink’. The ink contained resazurin dye, hydroxyl-ethyl cellulose, glycerol and distilled water. The degradation of the dye on the surface of the films was induced by a UVA (365 nm) light source with a photon flux of 1.09 × 10^15^ photons/cm^2^/s. The reduction in the concentration of the dye with time was monitored using UV-Vis spectroscopy. The formal quantum efficiency (FQE) and formal quantum yield (FQY) were calculated to enable easy comparison with literature.

The undoped TiO_2_ film was able to degrade resasurin at a rate of 1.4 × 10^11^ dye molecules/s/cm^2^, this was inferior to the 0.63 at.%, 1.65 at.% and 2.25 at.% W doped TiO_2_ films that had a degradation rate of 3.2 × 10^11^, 3.5 × 10^11^ and 2.4 × 10^11^ dye molecules/s/cm^2^ respectively. However at 4.47 at.% and 4.65 at.% doping levels the rates of degradation (7.9 × 10^10^ dye molecules/s/cm^2^ for both) was inferior to the undoped TiO_2_ film. Furthermore, compared to SGG Bioclean^TM^ self-cleaning glass, that was able to degrade resazurin dye at 1.2 × 10^11^ dye molecules/s/cm^2^ all the films excluding the 4.47 at.% and 4.65 at.% doped films were superior.

This shows that the presence of W in the TiO_2_ films does indeed enhance the photocatalytic properties but excess dopants can also be detrimental. The initial increase in the photocatalytic properties of the films with doping was most likely due to the increase in surface microstructure (and also due to the decrease in grain boundaries that are charge carrier recombination sites) due to larger, better shaped particles as observed from the SEM images. This facilitates a high surface area and more photoactive sites for the dye to be degraded during UVA illumination. With excess dopant concentrations (4.47 at% and 4.65 at.%) an increase in the surface area is off set by the presence of more photoinduced charge carrier recombination sites that in total reduce the photoactivity.

**Figure 7:**
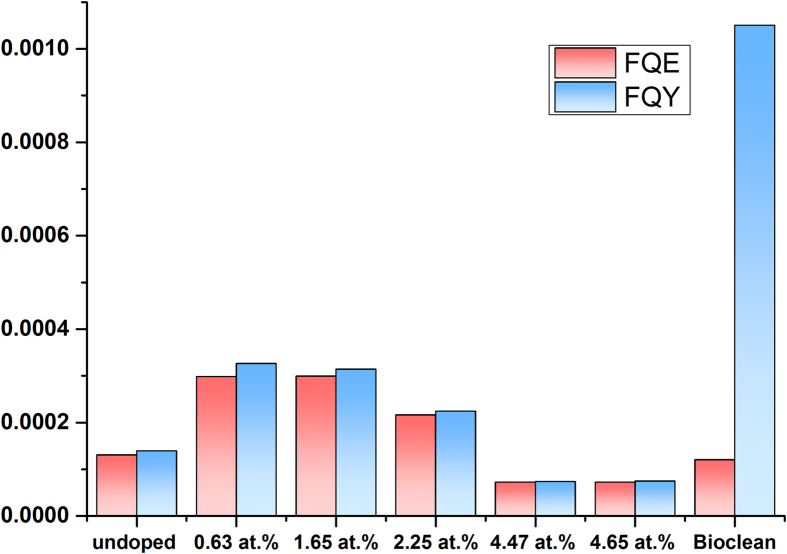
The graph shows the comparison of the FQE and FQY of the photodegradation of resazurin redox dye on the undoped and doped TiO_2_ films grown *via* AACVD. The FQE and FQY for an industry standard Bioclean^TM^ is also shown.

The formal quantum efficiency (FQE) and yield (FQY) for the films were calculated using the dye degradation rates, the UVA photon flux and UVA photon absorption for each of the films (Fig. 7). The 0.63 at.% and 1.65 at.% doped films showed the best results, with a FQE of 2.98 × 10^−4^ dye molecules per incident photon and FQY of 3.26 × 10^−4^ dye molecules per absorbed photon for the 0.63 at.% doped film and 2.99 × 10^−4^ dye molecules per incident photon (FQE) and 3.14 × 10^−4^ dye molecules per absorbed photon (FQY) for the 1.65 at.% doped film. This was more than double what was observed for the undoped TiO_2_ film grown under the same conditions. The principle reason for this was the more structured surface morphology observed for the doped films compared to the undoped. Furthermore, the FQEs of the 0.63 at.% and 1.65 at.% W doped films were also both more than double what was observed (1.2 × 10^−4^ dye molecules per incident photon) for SGG Bioclean^TM^. The FQY of SGG Bioclean^TM^ was however far superior with results an order of magnitude higher than the best preforming W doped TiO_2_ film.

Compared to literature, the W doped films display the same order of photocatalytic activity (FQE and FQY) relative to W doped TiO_2_ films studied by combinatorial APCVD^15^. Resazurin based redox dye test preformed on Pilkington Activ^TM^ self-cleaning glass by Mills *et al.* shows a FQE of 2.5 × 10^−4^ dye molecules/incident photon^26^. This is in the same order of magnitude as what was observed for the W doped TiO_2_ samples in this study, indicating the films are on par with industry standards.

## Conclusion

Transparent, electrically conductive and photocatalytically active thin films of W doped TiO_2_ films were synthesisied *via* a solution based CVD technique (AACVD). The structured morphology of the doped films combined with the good conductivites (optimum of 0.034 Ω.cm) and optical transparency in the visible region make them suitable candidates for electrodes in photovoltaic devices. Furthermore, the doped films displayed enhanced photocatalytic activity compared to the undoped TiO_2_ sample in the destruction of resazurin redox dye when illuminated by UVA radiation. In conclusion, this paper presents the single step formation of W doped TiO_2_ films with four desirable functional properties:

1. Transparent in the visible region
2. Photocatalytically active
3. Coloured
4. Electrically conducting

## Methods

### General Procedure

Depositions were carried out under nitrogen (99.99% from BOC). Precursors were placed in a glass bubbler and an aerosol mist was created using a piezoelectric device. All chemicals were procured from Aldrich and were utilised as recieved.

[Ti(OEt)_4_] (2 g, 8.8 mmol) was dissolved in toluene (25 ml) and [W(OEt)_5_] was added in dopant amounts (0 mol.% – 20 mol.%). The resulting solution was stirred for 30 minutes and then atomised. The precursor flow was kept at 1 L.min^−1^. The substrate temperature was kept at 500 °C. Deposition time was 45 minutes. After the deposition the bubblers were closed and the substrates were cooled under a flow of nitrogen.

At the end of the deposition the nitrogen flow through the aerosol was diverted and only nitrogen passed over the substrate. The glass substrate was allowed to cool with the graphite block to less than 100 °C before it was removed. Coated substrates were handled and stored in air. The coated glass substrate was cut into *ca.* 1 cm × 1 cm squares for subsequent analysis.

### Film Analysis

Powder X-ray diffraction (PXRD) was used to analyse the samples in a modified Bruker-Axs D8 diffractometer with parallel beam optics equipped with a PSD LynxEye silicon strip detector to collect diffracted X-ray photons. This instrument uses a Cu source for X-ray generation with CuKα_1_ and CuKα_2_ radiation of wavelengths 1.54056 Å and 1.54439 Å respectively, emitted with an intensity ratio of 2:1, a voltage of 40 kV and current of 30 mA. The incident beam angle was kept at 1° and the angular range of the patterns collected was 20° < 2θ < 66° with a step size of 0.05° counted at 0.5 s/sep.

Scanning Electron Microscopy (SEM) was performed to determine surface morphology and film thickness using a JEOL JSM-6301F Field Emission SEM at an accelerating voltage of 5 kV.

The W:Ti at.% composition was investigated via wavelength dispersive X-ray (WDX) analysis on a Phillips instrument. The average W:Ti % over four area scans (200 mm^2^) of each position analysed was taken.

X-ray photoelectron spectroscopy (XPS) was performed using a Thermo Scientific K-alpha photoelectron spectrometer using monochromatic Al-K_α_ radiation. Survey scans were collected in the range 0–1100 eV (binding energy) at a pass energy of 160 eV. Higher resolution scans were recorded for the principal peaks of Ti (2p), W (4f), O (1s), C (1s) and Si (2p) at a pass energy of 50 eV. Peak positions were calibrated to carbon (284.5 eV) and plotted using the CasaXPS software.

UV/Visible/near IR spectra were taken using a Perkin Elmer Fourier transform Lambda 950 UV/Vis spectrometer over a wavelength range of 300 nm to 2500 nm in transmission mode. The transmission spectra were taken against an air background.

Hall effect measurements were carried out using the van Der Pauw method to determine the sheet resistance, free carrier concentration (N) and mobility (μ). A square array of ohmic contacts arranged on 1 cm^2^ samples were then subjected to an input current of 1 mA and a calibrated magnetic field of 0.58 T. The transverse voltage was then measured. The measurement was repeated by reversing the direction of the magnetic field and the current.

Prior to the photocatalysis study, the samples were washed with distilled water, rinsed in isopropanol and irradiated for 30 minutes with UVA light to clean and activate the surface. The photocatalysis test involved spray coating the samples with an even layer of the resazurin based ‘intelligent ink’ (with modifications to the original recipe used by Mills *et al.*). The intelligent ink consisted of resazurin (40 mg) redox dye in an aqueous solution (40 mL) with glycerol (3 g) and hydroxyl-ethyl cellulose (0.45 g). The mixture was aged for 24 hours in a fridge (2–5 °C) and thoroughly mixed before use.

UVA light (flux = 1.01 × 10^15^ photons/cm^2^/s) was used to induce the photoreduction of the resazurin redox dye on the surface of the films and a UV-visible spectrometer was employed to monitor the degradation of the dye. Formal quantum efficiency (FQE) was calculated by dividing the rate of dye molecules destroyed (cm^−2^ s^−1^) by the photon flux (1.01 × 10^14^ photons cm^−2^ s^−1^). The formal quantum yield (FQY) was calculated by dividing the rate of dye molecules destroyed (cm^−2^ s^−1^) by the number of photons absorbed (cm^−2^ s^−1^) by the film. The photon flux and photon absorption for each film was determined using a UVX radiometer with a detector for 365 nm radiation attached.

## Additional Information

**How to cite this article**: Sathasivam, S. *et al.* Tungsten Doped TiO_2_ with Enhanced Photocatalytic and Optoelectrical Properties via Aerosol Assisted Chemical Vapor Deposition. *Sci. Rep.* **5**, 10952; doi: 10.1038/srep10952 (2015).

## Supplementary Material

Supplementary Information

## Figures and Tables

**Table 1:** The table shows the W concentration in the films as determined by WDX as well as the lattice parameter calculations for the undoped and W doped TiO_2_ films grown via AACVD at 500 °C.

W content in soln/ mol.%	W:Ti ratio in film / at.%	a / Å	c / Å	Unit cell Vol. / Å^3^	Vol. Expansion / %
0	—	3.7762(1)	9.431(1)	134.50(1)	0
2	0.63 : 29.59	3.785985(5)	9.464915(5)	135.67(2)	0.87
5	1.65 : 28.99	3.78213(9)	9.500(2)	135.89(3)	1.04
10	2.25 : 28.71	3.78287(6)	9.499(3)	135.93(4)	1.07
15	4.47 : 26.52	3.7811(8)	9.51(2)	136.03(2)	1.14
20	4.65 : 26.70	3.8009(5)	9.458(2)	136.64(3)	1.59

**Table 2:** The electrical properties of the undoped and doped TiO_2_ films grown via the AACVD reaction of Ti(OEt)_4_ and W(OEt)_6_ in toluene at 500 °C as determined by Hall effect measurements.

W content in soln/mo.l%	W:Ti ratio in film/at.%	Film thickness/μm	Sheet resistance/Ω sq^−1^	ρ/Ω.cm	μ/cm^2^/V.s	N/cm^−3^
0		—	—	—	—	—
2	0.63 : 29.59	1.3	4861	0.63	13.0	7.63 × 10^17^
5	1.65 : 28.99	0.8	3433	0.29	13.4	1.64 × 10^18^
10	2.25 : 28.71	2.8	212	0.034	14.9	1.23 × 10^19^
15	4.47 : 26.52	5.4	78	0.042	0.32	4.70 × 10^20^
20	4.65 : 26.70	4.1	154	0.063	0.049	2.02 × 10^21^
