# Supplementary material for: Tungsten Doped TiO2 with Enhanced Photocatalytic and Optoelectrical Properties via Aerosol Assisted Chemical Vapor Deposition
Source: Sci Rep. 2015 Jun 4;5:10952. doi: 10.1038/srep10952 (PMC4650706; doi:10.1038/srep10952)
Supplement: Supplementary Information [file srep10952-s1.doc]

**Tungsten Doped TiO_2_ with Enhanced Photocatalytic and Optoelectrical Properties via Aerosol Assisted Chemical Vapor Deposition**

Sanjayan Sathasivam*^1,2^*, Davinder S. Bhachu*^1^,* Yao Lu*^1^*, Nicholas Chadwick*^1^*, Shaeel A. Althabaiti*^3,4^*, Abdulrahman O. Alyoubi*^3,4^*, Sulaiman N. Basahel*^3,4^*, Claire J. Carmalt*^1^* and Ivan P. Parkin*^1^**

*****Corresponding author

^1^Materials Chemistry Centre, Department of Chemistry, University College London, 20 Gordon Street, London WC1H 0AJ, UK

Fax: (+44) 20-7679-7463

E-mail: [i.p.parkin@ucl.ac.uk](mailto:i.p.parkin@ucl.ac.uk)

^2^Bio Nano Consulting Ltd, The Gridiron Building, One St. Pancras Square, London N1C 4AG, UK

Fax: (+44) 20-7396-1056

E-mail: [info@bio-nano-consulting.com](mailto:info@bio-nano-consulting.com)

^3^Chemistry Department, King Abdulaziz University, Saudi Arabia

^4^Surface Chemistry and Catalytic Studies Group, King Abdulaziz University, Saudi Arabia

**Supporting information**


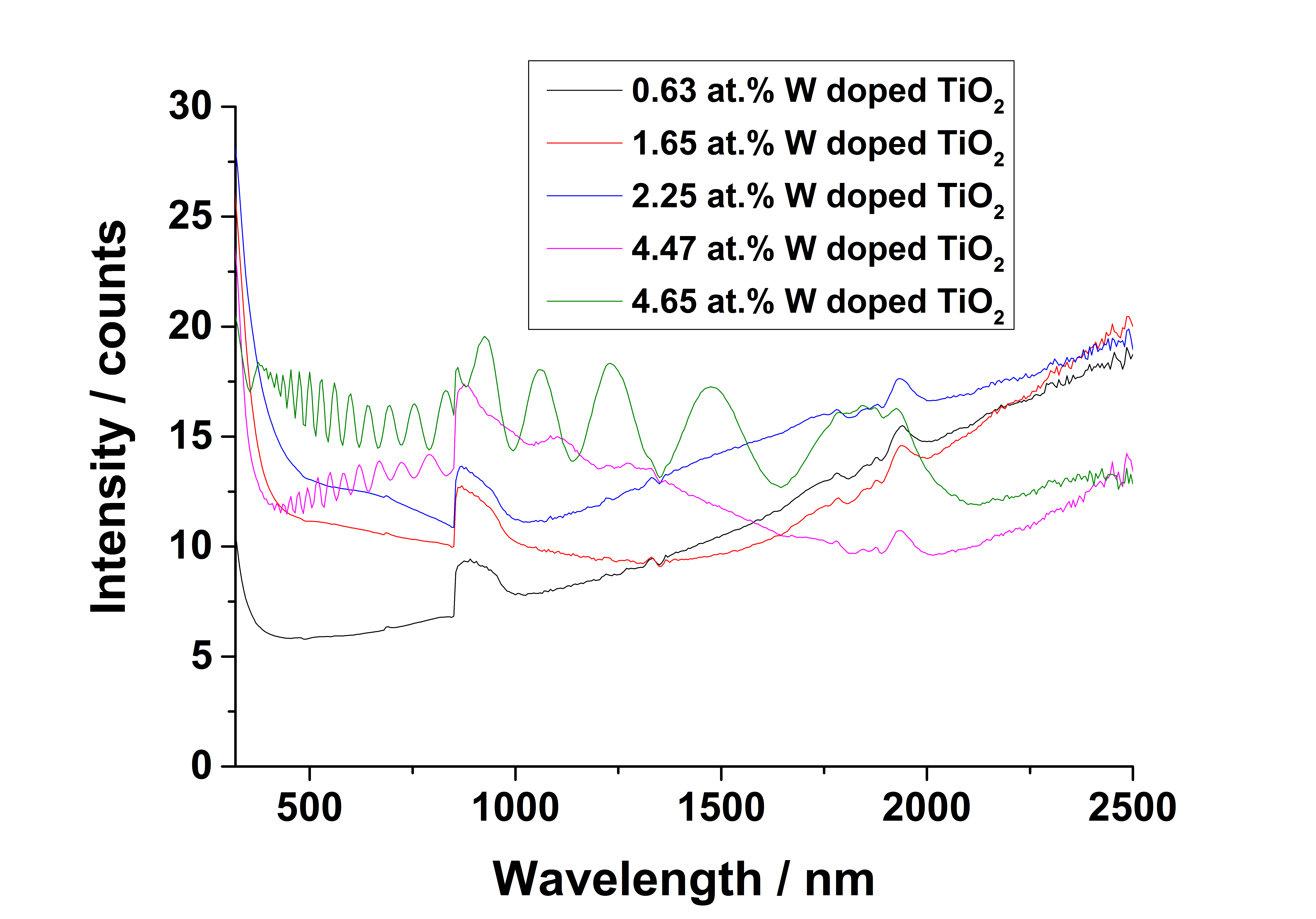


**Figure 1: The reflectance data for the W doped TiO_2_ films grown *via* AACVD**


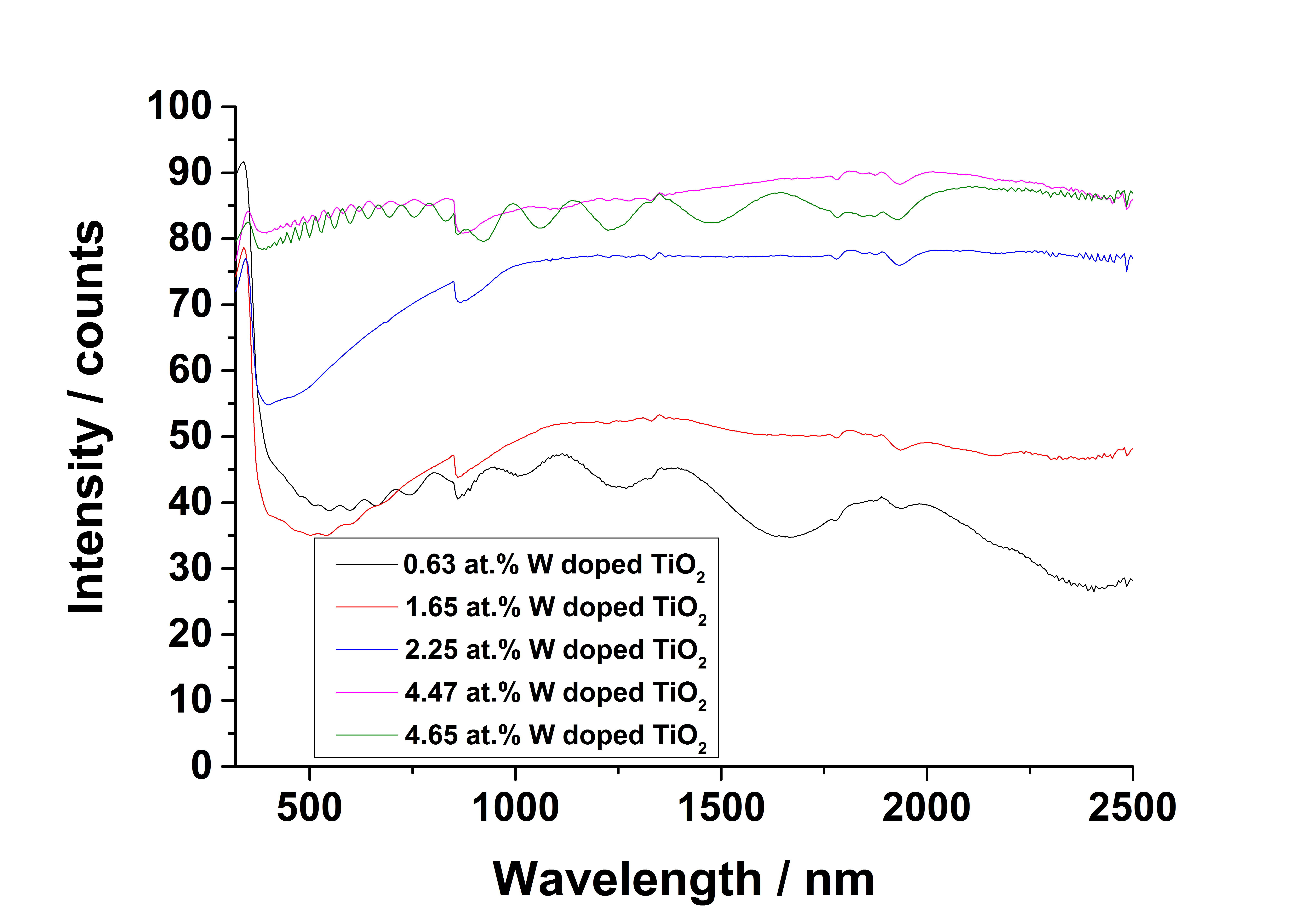


**Figure 2: The absorption data for the W doped TiO_2_ films grown *via* AACVD**
